# Health Information Related to Cardiovascular Diseases Broadcast on Chinese Television Health Programs

**DOI:** 10.3390/healthcare9070802

**Published:** 2021-06-25

**Authors:** Lei Yang, Jeroen Jansz

**Affiliations:** Erasmus Research Centre for Media, Communication and Culture, Erasmus School of History, Culture and Communication, Erasmus University Rotterdam, 3062 PA Rotterdam, The Netherlands; jansz@eshcc.eur.nl

**Keywords:** television health programs, health information, cardiovascular diseases, qualitative content analysis, traditional Chinese medicine, entertainment strategies

## Abstract

This study aims to add to the knowledge about information depicted in television health programs in China. Cardiovascular diseases (CVDs) are the leading cause of death in the country. The threat it poses is particularly notable among the Hui ethnic minority people, who have a higher prevalence of cardiovascular risk factors. Little research has been conducted thus far on content concerning CVDs in Chinese television health programs, and this study aims to fill this lacuna. Qualitative content analysis was conducted to analyze *The Doctor Is In* and *Health Body Light*. The results revealed that these two programs presented information about what CVDs and CVRFs are, how the former are treated, and what strategies are used to communicate health information. A new topic also stood out: discussions on how traditional Chinese medicine prevents and cures these diseases.

## 1. Introduction

Despite the great popularity of the internet, social media and apps in China, television (TV) remains a prominent source of information on health and well-being for many people [1,2]. Previous research has found that Chinese TV health programs present information on a variety of diseases and how to prevent them, with a common aim of improving their audience’s lifestyles and health awareness [3]. In this study, we focus on cardiovascular diseases (CVDs), because these are the leading cause of death in the Chinese population [4,5,6]. Little research has thus far been dedicated to examining how much and what CVD-related information is presented in these programs, and this study aims to fill that lacuna. As health communication also includes the issue of prevention, the decision was made also to study the representation of the five main cardiovascular risk factors (CVRFs): hypertension, diabetes, dyslipidemia, overweight/obesity, and smoking [4,5,6,7].

There are differences in the prevalence of CVRFs in the various ethnic groups in China, and previous data highlights the high prevalence of CVRFs among the Hui group [4] due to their culturally different lifestyles and eating habits [8]. In our own earlier research, our Hui participants expressed a strong and specific need for information about treating and preventing CVDs, which would help them to develop a lifestyle that reduces their likelihood of developing these conditions [9]. The Hui participants mentioned that they mainly used TV to gain CVD-related health information [2], but these participants also shared the difficulties they faced in obtaining health information [9,10], which could be an issue of access or availability. This study aims to fill this gap by investigating if and how CVD-related health information relevant to the Hui people is covered in Chinese TV health programs. This research is the first study to investigate what kinds of CVD-related health information is broadcast on Chinese TV programs and whether the Hui minority group is mentioned. We used qualitative content analysis to examine the content of multiple episodes of two popular TV health programs—*The Doctor Is In* and *Health Body Light*, which are, respectively, broadcast across China and in the Liaoning province.

## 2. Theoretical Framework

This study examines content related to CVDs shown on Chinese TV health programs. Diffusion theory provides a basis for legitimizing this focus. Diffusion theory can be seen as a repository collecting concepts from various social sciences concerned with the transfer of knowledge and spreading of information to the population [11]. Previous research has shown that diffusion theory helps emphasize mass media’s role in disseminating health innovations [11,12], for example, it has been used successfully in several studies including those on the use of alcohol, tobacco and illicit drugs [13]. Thus, diffusion theory helps the diffusion of health promotion via public education programs [13]. The current study is concerned with specific health content in two TV programs and is led by the first research question:

RQ1. What kinds of CVD-related health content are depicted in *The Doctor Is In* and *Health Body Light*?

### 2.1. The Ways TV Health Programs Broadcast Health Information

The depiction of health information can reinforce or hinder its impact on knowledge, attitudes, and behaviors [14]. It has also been shown that how such information is presented in mass mediated health campaigns is particularly important for attracting the attention of viewers [15]. According to the multimedia principle [16], an audience’s comprehension increases when textual information is combined with pictures, because “a picture is worth a thousand words” (p. 117, [16]). Different formats have been used to communicate health information on TV; medical dramas, comedies and soap operas, for example, have been used in the entertainment–education tradition [14,17,18,19], while entertaining elements like quizzes or games have been integrated into programs that primarily focus on communicating information [17]. These different approaches provide the background for our second research question:

RQ2. In what ways do *The Doctor Is In* and *Health Body Light* broadcast health information related to CVDs?

### 2.2. The Chinese Hui Ethnic Minority and Cardiovascular Diseases

In China, there are 55 ethnic minority groups and the Han majority group [20]. Among these 55 minority groups, there are 10 Islamic groups which include the Chinese Hui minority group [21]. “Big distribution, small concentration” is the character of the Hui group, because they live across the so-called Hui autonomous and Hui nonautonomous areas [22]. Compared to the Hui people in the Hui autonomous areas in Northwest China, the Hui in the East of China receive less attention from mediated sources [9]. Therefore, we chose to focus on the Hui people in Shenyang City.

Our major reason for investigating the depiction of CVD-related information on Chinese TV is the high prevalence of CVDs and CVRFs among the Hui minority [23]. Even though the Hui share a language, customs and culture with the Han, which is the Chinese ethnic majority [24], they also have their own ethnic culture. Their Islamic faith also results in different dietary habits. In addition, they also have special health circumstances, especially with respect to CVDs and CVRFs.

Previous research that has built on the culture-centered approach (CCA) shows that health communication tends to focus on dominant groups in multicultural and multiethnic societies, which often means that the health needs of minorities are ignored [25,26,27,28]. In our recent survey of the Hui, we established that they did indeed have specific needs with respect to health information [9]. Therefore, our third research question is:

RQ3. What health content specifically related to Hui people is included in the two TV programs?

## 3. Methods

Our research questions focus on the health content, in particular the text, communicated in two Chinese TV health programs: *The Doctor Is In* and *Health Body Light*. We used qualitative content analysis to answer the research questions, following previous studies concerning health-related media coverage (e.g., Glenn et al., 2012 [29]). Content analysis provides appropriate tools for analyzing the content of TV programs, as it enables valid and replicable inferences to be drawn from what is shown on the screen [30].

### 3.1. Case Selection

National and local TV stations in China both broadcast health programs. The first program examined in this study, *The Doctor Is In*, is transmitted by the national TV station, China Central Television (CCTV), while the second, *Health Body Light*, is aired by the local station, LRTV-City. Both programs are targeted at the general public and are set in the indoor studios of the respective stations.

This study is part of a larger project carried out in Shenyang City from 19 December 2016 to 9 February 2017. A survey was conducted in that period to investigate the Hui’s access to and evaluation of health information concerning CVDs, available on various communication channels and sources [2]. Focus groups were then used to explore the particular needs the Hui had relating to these diseases [9,10]. The current research concentrates on TV programs that were broadcast in the same period, including 90 episodes of *The Doctor Is In* and 89 of *Health Body Light*. Chinese spring festival holidays meant that six of the 89 episodes of the latter were repeats of programs aired in January 2017, and these were therefore removed from our corpus.

Relevance sampling was used in the study, with the aim being to select all the textual units that contributed to answering the research questions [30]. The two TV programs examined are also available online. The lead researcher watched all the episodes to identify those concerning CVDs, resulting in a corpus of 13 from *The Doctor Is In* and 12 from *Health Body Light.* These episodes were downloaded in the MP4 format and stored. We only analyzed the CVD-related content, meaning that messages shown in advertisements, public service announcements, and promotions of other TV programs were excluded.

### 3.2. Analysis

Our unit of analysis was an individual episode of a program, which included the introduction, main content, and the ending. Each episode of *The Doctor Is In* and *Health Body Light* is around 37 ± 2 and 28 ± 1 min long, respectively. We examined what was presented and discussed in relation to CVDs and CVRFs in the selected episodes. The coding unit of analysis was a sequence. According to Iedema [31]: “Sequences will comprise a range of contiguous scenes which are linked not on the basis of space and time continuity, but on the basis of a thematic or logical continuity” (page 189). In our analysis, the selected episodes were each divided into sequences, resulting in a total of 104.

We then hand-coded the episodes. Thematic analysis was used to scrutinize the sequences, as this is a systematic procedure with enough flexibility to provide (theoretical) freedom in the interpretation [32]. An inductive approach was used, enabling us to capture the main themes emerging from the data [33]. Because this was an explorative study with limited resources, we employed an efficient coding procedure in which all episodes were hand-coded by the researcher. To obtain an indication of the quality of the coding, 20% of the episodes were also coded by a second independent coder. After coding this selection, the codes of the two coders were compared. This resulted in 80% similarity, which gave us confidence in the quality of the coding. The coding proceeded stepwise, with the first step being ‘open coding’, in which the coder watched all the sequences and coded the themes inductively. The coding mainly focused on: (1) the kinds of CVD-related information transmitted: e.g., prevention, the use of medicines; (2) the ways this information was conveyed: e.g., dramas, cartoons; and (3) whether the Hui were targeted, e.g., where and if they were mentioned and in what way. Axial coding was then used to extract themes from the content, enabling us to outline the kinds of CVD-related health information covered and the ways it was conveyed. Finally, we conducted selective coding by choosing representative quotes from different sequences in the programs. The results of the analyses provided an overall description of what and how CVD-related health information is depicted in these TV programs.

## 4. Results

### 4.1. Brief Descriptions of the Two Programs’ Formats

The two programs investigated included various amounts of CVD-related health information. *The Doctor Is In* generally started with a brief live drama acted out by two special guests to highlight the subject-matter of an episode. The female host then appeared to introduce the topic and the two guests sat on the stage in front of the audience. In what followed, the host asked the audience topic-related questions and welcomed an expert, who answered these and provided medical explanations. The two guests who had acted in the live drama asked the expert relevant follow-up questions based on their experiences to facilitate further clarification. There were also interactions between the host, the guests and the expert that made the explanations clear and easy for the audience to understand. Sometimes, the audience had the opportunity to talk to the expert. An assistant was used when the expert needed to demonstrate practical medical knowledge. At the end of the program, the host summarized what had been covered in that day’s episode.

Most of the episodes of *Health Body Light* started with a card located in a ‘love mailbox’ related to the topic to be covered, which was read by one male and one female host. There was then a brief video of people outside the studio asking questions related to this theme. The assistant host, who sat with the live audience, introduced the subject matter and welcomed an expert guest. After a brief discussion between the expert and the two hosts, the former started to answer questions and provided medical explanations. There were also interactions between the live audience, the hosts and the expert. In some episodes, an assistant in cartoon dress turned up to help the expert distribute something to drink that was related to the topic or to show samples of medicines to the audience. At the end of an episode, the two hosts and the expert used three to five short sentences to summarize the important information discussed.

Our thematic analyses of the content showed that the two TV programs mainly depicted health information about three related topics: (1) what CVDs and CVRFs are; (2) how CVDs are treated; and (3) what strategies are used to communicate CVD-related health information. A fourth topic also stood out: how traditional Chinese medicine prevents and cures CVDs. Despite the high prevalence of CVDs and CVRFs among the Hui, there was no information specifically targeted at this minority in our corpus.

### 4.2. What Kinds of CVD-Related Health Content Are Depicted in The Doctor Is In and Health Body Light?

#### 4.2.1. What Are CVDs and CVRFs?

To answer our first research question about the kinds of CVD-related health content depicted in *The Doctor Is In* and *Health Body Light*, almost all of the episodes of the two programs we examined (23 of 25) contained general health information related to CVDs. The characteristics and symptoms of the diseases were described, and then the causes and high-risk populations were identified. The dangerous consequences of CVDs, the relationships between them and other diseases, and between CVRFs and lifestyles were also discussed. The general information was usually presented by the experts, who were depicted in a way that affirmed their professionalism and credibility; for example, when they appeared on the set, their academic titles were shown on the screen immediately.

The experts present in both programs provided information to counter any misunderstandings that people may have about CVDs. The 18 December 2016 episode of *Health Body Light* was a typical example. Its subject matter concerned overweight/obesity. The expert, Dr. Fan (Director of Chinese Nutrition Society), explained what this was, and then clarified the differences between being overweight and being strong, as it was claimed that many people confused the two. She then also corrected other misunderstandings; for example, she stated: “Many people think that overweight people’s intake of nutrients is more than needed, but that is not right”.

The experts who appeared on the two programs explained the causes of CVDs and promoted dietary and lifestyle-related health behaviors that could prevent these conditions or reduce CVRFs. The episode of *Health Body Light* on 2 January 2017 concerned hypertension. Dr. Fan explained that eating at irregular intervals was one of the causes of this, and then promoted a few healthy dietary habits (e.g., eating fruit).

The episodes in our corpus contained health information that people could use to identify the symptoms of CVDs by themselves. The experts also stressed that if symptoms were present, a doctor should be seen as soon as possible. In the 16 January 2017 episode of *The Doctor Is In*, the host introduced its topic by asking the audience the following question: “What signs should you be aware of regarding myocardial infarction?” The expert, Prof. Yang (Chief physician at the Beijing Chaoyang Hospital, Capital Medical University), explained each symptom of the condition in detail and, at the end of the program, suggested that viewers should go to hospital or call for an ambulance without delay if they experienced any of them.

Overall, both programs contained health information about what CVDs and CVRFs are. The experts also corrected any misunderstandings, promoted healthy behaviors for disease prevention, and helped the audience to recognize any symptoms to ensure they were treated quickly.

#### 4.2.2. How Are CVDs Treated?

The treatment of CVDs can be viewed as solutions to health issues. In the different episodes of the two programs, treatments were mainly discussed in the context of giving first aid and the use of medicines. Thus, this is also an important part of CVD-related health content that is depicted in *The Doctor Is In* and *Health Body Light*.

The issue of delivering first aid was prominent in both programs. The hosts stressed the importance of having first-aid skills, as these can save lives in medical emergencies, particularly because patients with CVDs are high risk. The experts also demonstrated how to provide first aid. On the 6th December 2016 episode of *The Doctor Is In*, the expert, Prof. Li (Director of the Cardiovascular Center, Beijing Friendship Hospital, Capital Medical University), explained what to do if someone suddenly collapsed and stopped breathing. Two special guests were invited to perform cardiopulmonary resuscitation (CPR) on a model of the human body, but did so incorrectly. Thereafter, Prof. Li’s assistant demonstrated the right way to provide this type of first aid.

In our corpus of 25 episodes, information on medicines related to CVDs is commonly included. This mainly focused on: (1) how to take the medications correctly; (2) their functions; (3) the consequences of misusing them; and (4) which ones to use in emergencies. Both programs covered the important role medicines play. The experts highlighted that many patients did not take them correctly and explained why particular methods were right or wrong. The 18 December 2016 episode of *The Doctor Is In* contained a typical example: in a live scene of a dinner, a mom and son ate food containing a lot of sugar and fat. The son then said that he would take another pill containing hypoglycemic agents to counteract the extra sugar he had eaten that day. The live audience echoed that they did the same thing by increasing the dose of the medications they had been prescribed. Prof. Li (Chief Physician at Fuwai Hospital, Chinese Academy of Medical Sciences) then explained why this was wrong and what the consequences were, before describing the proper way of taking these drugs. In doing so, he not only corrected the audience’s misunderstandings about using medicines, but also provided instructions on how to take them properly.

Another interesting sub-theme that emerged from our analysis concerned the discussion of the credibility of health information obtained from social media platforms such as WeChat. Each day, approximately 570 million users, 93% of whom live in first-tier cities in China, log in to their WeChat accounts [34], and the platform is treated by Chinese people as an important way to acquire health information [34]. The 1st December 2016 episode of *Health Body Light* concerned the medical effects of okra water. A member of the live audience in the studio shared that she had learned from WeChat that drinking this water could lower blood sugar levels and even eliminate the need for hypoglycemic agents, although she was not sure whether this was true. A nutrition expert, Director Wang (Director of the Nutrition Department of Dalian City Central Hospital), corrected this misunderstanding, stating that okra water is helpful but could not replace these medications: “As long as you don’t stop taking hypoglycemic agents, then it (drinking okra water) is fine.” In this episode, other members of the live audience revealed that they also obtained and shared health information within their social networks on WeChat. As an example, a young man’s friend on the platform told him that soda water could kill cancer cells, causing him to buy a few bottles. The two programs also have their own WeChat official accounts, which implies that they intersect TV broadcasting with social media. The contents of these accounts were not, however, analyzed in this study.

#### 4.2.3. How Does Traditional Chinese Medicine Prevent and Cure CVDs?

The episodes in our corpus provided information about traditional Chinese medicine in relation to CVDs, in particular its role in preventing them and relieving their symptoms. Thus, traditional Chinese medicine is also an important part of CVD-related health content that is depicted in *The Doctor Is In* and *Health Body Light*.

Three aspects of traditional Chinese medicine were covered in both programs: (1) acupressure; (2) making food with traditional Chinese medical ingredients; and (3) scraping. In relation to the first, Prof. Jia (from Beijing University of Chinese Medicine) explained that patients can press specific acupoints to prevent diseases, relieve symptoms, or save themselves in emergencies. The experts showed the audience how to identify and press their acupoints, which they explained was necessary because many patients did not know how to do this properly. For example, in the 4 January 2017 episode of *The Doctor Is In*, Prof. Jia showed the audience two important acupoints—Zhiyang and Jiuwei—using a picture that was clearly marked, then he commented: “Pressing these two acupoints can help people relieve their discomfort and cure themselves of angina pectoris.”

The experts also presented information about preparing specific kinds of food (e.g., soup) that use traditional Chinese medical ingredients to prevent and relieve the symptoms of CVDs. Five episodes of *Health Body Light* introduced home-made drinks and food produced using such elements as a form of adjuvant therapy. On *Health Body Light*, the episode on 3 January 2017 focused on cardio issues. The expert, Dr. Wu (Chief physician at Dongzhimen Hospital, Beijing University of Chinese Medicine), explained how to make ‘Happiness tea’ with traditional ingredients that could make the heart feel better and relieve symptoms.

Scraping was the third aspect of traditional Chinese medicine discussed in the two programs. Dr. Wang (Deputy Chief Physician at the Acupuncture and Moxibustion Hospital of China Academy of Chinese Medical Sciences) was the expert on the 12 February 2017 episode of *The Doctor Is In*. She argued that scraping had become more popular in China, but some had doubts about its effectiveness. She then explained that many people carry it out incorrectly, which could cause serious side effects, before showing the audience the proper way to relieve hypertension by scraping a particular acupoint. She also explained that the strength of scraping and the time of its application matter a great deal for its efficacy.

The experts who appeared on these two programs emphasized the importance of applying interventions based on traditional Chinese medicine properly, as they were convinced that patients often did not do so.

### 4.3. In What Ways Do The Doctor Is In and Health Body Light Broadcast Health Information Related to CVDs?

To answer our second research question, our analyses revealed that the two programs in our study used simple entertainment strategies to disseminate health information. Different approaches were identified in the 25 episodes examined (see Table 1).

Cartoons were used as a tool in both programs to help the experts explain medical phenomena or theories. For example, in the 16 December 2016 episode of *The Doctor Is In*, after the experts had verbally explained the principles of using medicines to lower blood pressure, the host said: “Some audience members may not understand what you’ve just explained, so let’s use tools to explain it to them again, okay?” The cardiovascular specialist, Prof. Xu (Chief Physician at Fuwai Cardiovascular Diseases Hospital, Chinese Academy of Medical Sciences), used pictures of a rabbit with a carrot, a turtle with a fish, and a giraffe with some leaves to explain why it is necessary to use medicines in the correct manner. The cartoons in this episode clearly aimed to explain complex health information in an easy-to-understand way.

Of the 25 episodes we examined, five from *The Doctor Is In* and one from *Health Body Light* incorporated live dramas to present health information. In some cases, these were used to introduce the program’s topic, but also made the program more entertaining. The 15 January 2017 episode of *The Doctor Is In* started with a live drama played out by the host and two guests, which brought laughter from the live audience. After the drama, the expert, Dr. Zhang (Deputy Chief Physician at the Department of Traditional Chinese Medicine of the Wangjing Hospital of Chinese Academy of Medical Sciences), explained that the traditional Chinese medicine ‘an gong niu huang wan’ can only be used in cerebral infarctions, hemorrhages or heart attacks with specific symptoms, and not in the emergency case shown in the drama.

Both programs included quizzes to engage the participants with the health information. When the live audience, special guests and hosts took part in these, they also disclosed their health conditions and shared their opinions. The 6 December 2016 episode of The Doctor Is In started with a health knowledge quiz between a blue and a red team. The live audience and special guests were divided into two teams and the expert was the judge. The two special guests were celebrities and the team captains. After the two guests provided answers on behalf of each team, the expert judge gave the right answer and explained the medical issue in more detail. During the quiz, the guest captains of the two teams made fun of each other, which caused much laughter in the audience.

## 5. Discussion

This is an exploratory study to focus on CVD-related health content in Chinese TV health programs. Previous research has shown that misunderstandings of health information exist in the US, e.g., concerning the use of medicines [35,36,37]. The situation in China seems to be similar, because many adults in the country are unable to fully understand verbal and written health information in a healthcare setting [38]. In our research, the experts appearing on the two programs often assumed that people generally misunderstood information related to CVDs, and both therefore strongly emphasized the need to develop an individual’s expertise.

Traditional Chinese medicine emerged as an important theme. China has two kinds of medical system, traditional Chinese and Western, which are different in how they conceptualize health and deal with illness. Traditional Chinese medicine is rooted in Chinese philosophy and is an important part of the Chinese culture [39]. Traditional Chinese medical treatments make use of theories passed down through generations, while modern Western medicine prescribes treatments for specific diseases based on a patient’s physiology [39]. Many of the episodes we examined presented a great deal of information about using traditional Chinese medicine to prevent and cure CVDs, as an alternative to the Western approach. This finding was consistent with previous research, which found that certain concepts of traditional Chinese medicine may help to improve its Western counterpart [40]. Nowadays, more people in China realize that the two medical systems may have their own merits and the researchers suggest that they should take advantage of each other’s strong points [40].

The two TV programs used basic entertainment strategies to make them more amusing and appealing. Cartoons were used in combination with written text in an attempt to explain medical facts in an easy-to-understand way. This is logical from the perspective of the multimedia principle, which holds that individuals will comprehend information better when words and pictures are presented together [16]. Other strategies, such as quizzes, also played a role in making the programs entertaining. In addition, we found that live audiences in the studio brought topics from WeChat to the floor, which illustrates how current social and traditional media often merge [41]. Health promotors could thus take advantage of this to convey health information to the public.

This study has several limitations. First, it is difficult to make generalizations across other health programs. A large-scale study examining a variety of Chinese TV health programs is therefore needed in the future. Additionally, this research focuses on one national and one local health program. Accordingly, future studies could be conducted to make comparative analyses of more national and local programs to investigate their differences in terms of the health information they disseminate. Second, the current study mainly focused on the text used in the programs, which may be seen as a limitation by some researchers. Although the visuals or comments of the audience also communicated meanings, text was prioritized because it enables an audience’s interpretations to be better understood [42], which was the goal of this research.

Despite the limitations, our findings have implications for future health communication in China. Previous research has shown that the Hui have the highest prevalence of hypertension [4], and both programs presented a substantial amount of information related to this condition. However, it was surprising to find that neither program included culturally or ethnically specific information for the Hui. As a consequence, health programs in the future should rectify this to ensure that the health needs of this minority group are taken into account.

## 6. Conclusions

This research only examined the kinds of CVD-related health information depicted in the selected health programs on Chinese TV, and if this content is related to the Hui minority. The scientific–medical validity of the content of the information was not studied in this project. In response to the first research question, the results revealed that both programs: contained a great deal of health information concerning what CVDs and CVRFs are; discussed how to clarify relevant misunderstandings; promoted healthy behaviors for prevention purposes; and helped people to recognize symptoms to ensure they are treated in time. The two programs mainly presented information about treatment for CVDs in the contexts of the use of medicines and first aid. In addition, a few episodes of both programs focused on traditional Chinese medicine, which led us to conclude that this plays an important role in China’s medical systems.

The second research question examined how the two programs depicted health information related to CVDs. Overall, the communication styles were similar, with an emphasis on serious advice, but both also included entertainment elements such as cartoons and quizzes.

Our third research question concerned how CVD-related health content was targeted at the Hui minority. It was found that, even though the Hui have a high prevalence of CVRFs and CVDs, no health information was specifically targeted at them in the corpus we analyzed.

## Figures and Tables

**Table 1:** Entertainment Strategies Used to Disseminate Health Information Related to CVDs.

Strategies	Number of Episodes
Cartoons	17
Live dramas	6
Quizzes	5

*Note*. Some episodes may contain multiple instances. Each instance is counted only once in each episode.

## Data Availability

The data of this article is concerned with two TV programs. The first program named *The Doctor Is In* is transmitted by the national TV station, China Central Television (CCTV), while the second program called *Health Body Light* is aired by the local station, LRTV-City. In this article, we included 90 episodes of *The Doctor Is In* and 89 of *Health Body Light* that were broadcast from 19 December 2016 to 9 February 2017. These episodes were public online when we conducted this study. These episodes were downloaded in the MP4 format and stored by the corresponding author.

## Notes

All quotes were translated as literal as possible from Mandarin to English.
